# Practical Notes on Diagnosis and Treatment

**Published:** 1907-01-19

**Authors:** 


### Practical Notes on Diagnosis and Treatment.

**Abdominal Conditions in Enteric Fever.**

The absence of abdominal distension and tenderness in enteric fever would seem to indicate the absence of extensive or deep-seated ulceration in the agminated glands. Sir Wm. Gull, in a doubtful case of typhoid, used to place great stress on the distension of the abdomen, as being one of the most trustworthy facts in making a diagnosis.—*Dr. G. H. Whipham.*

**Guaiacol in Enteric Fever.**

Dr. B. M. Baker recommends guaiacol in cases of enteric fever. He finds that it reduces the temperature and pulse, checks diarrhœa, and gets rid of tympanites. The dose he advises is five minims thrice daily.

**Otitis Media and Bathing.**

An occasional cause of acute otitis media is *frequent bathing*, especially the ducking of the head in water and allowing the water to get into the auditory canal, and to rest against the tympanum without thoroughly drying the ears.—*Mr. Henry Morris.*

**Olive Oil in Lead Poisoning.**

Olive oil in large doses, 5 or 6 ounces, will, it is said, promptly cure lead colic, and the same medicine given in 2-ounce doses in the 24 hours, has a beneficial action in the other symptoms of plumbism.

**Tonsillitis.**

The following is recommended as a prophylactic in patients liable to attacks of tonsillitis:—Oil of peppermint 8 drops, carbolic acid 1 drachm, rectified spirit 2 drachms. Ten drops added to a cup of warm water and used as a gargle night and morning.

**Bladder Weakness.**

In women who have had children there is a form of bladder irritation which is, perhaps, due to a slight prolapse of the pelvic floor, not enough, however, to be detected on examination. The chief symptom is the constant dribbling of small quantities of urine during the day. The patient is not disturbed while recumbent. Escape of urine is often promoted in such patients by coughing. This kind of bladder trouble is benefited by strychnine and ergot.—*Dr. G. E. Herman.*

**Chloride of Ethyl in Neuralgias.**

The local application of chloride of ethyl spray has sometimes not only relieved, but also cured some forms of neuralgia. Hemicrania, lumbago, and supra-orbital neuralgia have all been cured in this way.

**Creasote in Phthisis.**

For the success of creasote in phthisis pulmonalis it is essential (1) that the creasote should be absolutely pure; (2) that it be taken *immediately* after meals. The creasote should be given in gelatine capsules, each containing one minim. Begin with one capsule thrice daily, and gradually increase the dose until the patient is taking 15 minims of pure creasote thrice daily.—*Sir Felix Semon.*

**Local Applications in Inoperable Breast Cancer.**

Any lotion or ointment containing morphine is suitable, but I know few more soothing applications to an incurable breast cancer than the old-fashioned conium poultice. This may be made from the fresh leaves, or ½ ounce of the succus conii may be added to an ordinary breast poultice.—*Mr. Marmaduke Shield.*

**Garlic in Fœtid Expectoration.**

I have found garlic of distinct service in cases of dilated bronchi with fœtid expectoration. I have usually given the garlic in the food. A "clove of garlic" has been chopped up and boiled with the beef-tea, and only very few patients have been unable to take it in this way.—*Dr. Vivian Poore.*

**Renal Colic.**

Renal colic does not only occur as the result of the passage of a renal stone along the ureter, but other substances, such as blood-clots, mucus clumps, tubercular sloughs, hydatid cysts, and detached pieces of renal growth are able to evoke the same colicky pain and distress, though perhaps in a less degree.—*Mr. Hurry Fenwick.*

**Epilepsy.**

In cases of epilepsy, where the bromides fail, the following is sometimes successful: Zinc oxide, ½ grain; extract of belladonna, ½ grain; powdered valerian root, 15 grains. Take this in the form of a powder three times daily. The dose of the zinc oxide may be gradually increased until it reaches 5 grains thrice daily.

**Hypodermic Injection of Mercury.**

In cases of syphilis of the nervous system, in order to get the patient promptly under mercury the medicine should be given hypodermically. Inject once a day deep into the muscles of the gluteal region one-eighth of a grain of perchloride of mercury dissolved in five minims of water. If the syringe is clean there is no need to fear any bad result.—*Dr. Hale White.*

**Local Application in Acute Rheumatism.**

Professor Bourget concludes that salicylic acid is rapidly absorbed through the skin, and that the absorption is increased when the drug is mixed with a fatty basis. He therefore applies the following ointment to the affected joints in cases of acute rheumatism. Salicylic acid, 1 part; lanoline, 1 part; oil of turpentine, 1 part; prepared lard, 10 parts. To be smeared on without rubbing, and the joint to be then covered with flannel.

**Trional.**

According to Dr. Bryer, men need much larger doses of trional than women. In a man the commencing trial dose should be 17 to 25 grains, whilst with a woman 12 to 15 is sufficient.

**Osteotomy in Rachitic Deformity.**

I do not think that osteotomy ought ever to be done under the age of six years, for a very large proportion of rachitic deformities disappear as the child gets older.—*Mr. H. H. Cluton.*

